# Effects of Baneh (*Pistacia atlantica*) Gum on Human Breast Cancer Cell Line (MCF-7) and Its Interaction with Anticancer Drug Doxorubicin

**DOI:** 10.22037/ijpr.2019.1100853

**Published:** 2019

**Authors:** Hamzeh Pasban-Aliabadi, Vahid sobhani, Saeed Esmaeili-Mahani, Hamid Najafipour, Alireza Askari, Hamidreza Jalalian

**Affiliations:** a *Exercise Physiology Research Center, life style institute, Baqiyatallah University of Medical Science, Tehran, Iran. *; b *Department of Biology, Faculty of Sciences, Shahid Bahonar University of Kerman. Kerman, Iran. *; c *Physiology Research Center and Department of Physiology and Pharmacology, Afzalipour Medical Faculty, Kerman University of Medical Sciences. Kerman, Iran. *; d *Department of Hematology and Oncology, Baqiyatallah University of Medical Sciences, Tehran, Iran.*

**Keywords:** Baneh gum, Anti-apoptotic, Anti-proliferative, MCF-7 and doxorubicin

## Abstract

*Pistacia atlantica* is one of the species of *Anacardiaceae* that grows in the wild in different regions of Iran. Traditionally, *anacardiaceae* family has antibacterial, fungicidal, and cytotoxic properties. Therefore, the present study was designed to investigate the possible cytotoxic and anti-proliferative properties of Baneh gum. Cytotoxicity of the plant gum was determined using MTT assay on MCF-7 human breast cancer cells. The cellular makers of apoptosis (caspase3 and P53) and cell proliferation (Cyclin-D1) were evaluated by western blotting. Doxorubicin was used as anticancer control drug in combination treatment. The result showed that Baneh gum (100 µg/mL) significantly induced cell damage, activated caspase3, and increased P53 protein level. In addition, Cyclin-D1 was significantly decreased in gum-incubated cells. Furthermore, combination treatment of cells with Baneh gum (25 µg/mL) and doxorubicin (200 nM) produced a significant cytotoxic effect as compared to each drug alone. In conclusion, Baneh gum (100 µg/mL) has a potential pro-apoptotic/anti-proliferative property against human breast cancer cells and its combination with doxorubicin in low doses may induce cell death effectively and be a potent modality to treat this type of cancer.

## Introduction

Cancer is a fetal disease with no complete therapy or cure, so it is critical to find beneficial solution to defeat this public health menace. Almost all chemotherapic drugs have unwanted and serious side effects. The development of resistance to chemotherapic drugs is a common clinical problem in the treatment of various cancers. In addition, common cytotoxic therapies primarily target rapidly dividing cells including malignant cells as well as certain normal cells, leading to significant morbidity and limited clinical benefits of troubled patients. Combination therapy is a useful method for the reduction of anticancer drug doses and subsequent side effects. 

 Breast cancer is common well known cancer and is the main death reason of cancer in woman throughout the world. Each year over 1 million women are diagnosed with breast cancer and nearly half of them die (1). At least one in nine women develops breast cancer at some stages in their life. Currently, there is an urgent need for improvements in detection, diagnosis, and treatment of breast cancer. Unfortunately, the current classical treatments (surgery, chemotherapy, and/or radiotherapy) are impeded by side effects most importantly development of tumor resistant, loss of appetite, nausea and vomiting, weakness and fatigue, mouth soreness, hair loss, weight gain, premature menopause, lowered resistance to infections, bleeding, and gastrointestinal illness (2).Therefore, finding novel and effective therapies to prevent the development of breast cancer and lower mortality rates related to it, is a current scientific challenge. 

Over 7,000 species of plants grow in Iran among them 1000 species are estimated to have medicinal effects (3). The presence of various climatic con­ditions and ecological factors provide an environment in which numerous varieties of plants grow in differ­ent regions of the country (4). The genus Pistacia belongs to the family of *Anacardiaceae*, and among 15 known species of pistachios. The *Psitacia* *atlantica* with the local name of Baneh has three sub-species: *mutica*, *kurdica* and *cabulica* (5). Baneh has been used in folk medicine for treating eczema, throat infection, renal stone, and asthma (6). It has been demonstrated that Baneh has Pro-apoptotic and Anti-proliferative effect on cancerous situation (7-9). The antioxidant (10), antimutagenic (11), antimicrobial and antiviral (12), anti-inflammatory (13, 14), antidiabetic (15), antitumor (16), anti-hepatitis (15), anti-atherosclerosis (17), and anti-cholinesterase (18) effects of Baneh have been reported. Previous studies used other parts (stem, leave, pericarp and etc.) or different spesies (*P. vera*, *P. atlantica*, *P.terebinthus*,* P. khinjuk*, and *P. lentiscus*) or subspecies of baneh (*cabulica* and *mutica*) instead of *piatatia atlantica sub *

*kurdica*. 

Despite numerous reports regarding cytotoxic effects of baneh, the effects of *pistacia atlantica* sub kurdica gum alone and along with doxorubicin on breast cancer cell line have not been yet reported. So, we decided to evalu­ate the cytotoxic effect of baneh gum on breast cancer cell line and its combinational therapy with doxorubicin.

## Experimental

*Materials*

Cell culture reagents, penicillin-streptomycin solution, trypsin EDTA, and fetal bovine serum (FBS) were obtained from Biosera Co. (East Sussex, UK). Culture flasks and dishes were acquired from SPL Lifesciences Inc. (Gyeonggi-Do, South Korea). 3- [4, 5-Dimethyl-2-thiazolyl]-2, 5-diphenyl-2-tetrazolium bromide (MTT), doxorubicin and primary monoclonal anti-𝛽-actin antibody were purchased from Sigma (St. Louis, MI, USA). Primary monoclonal anti-caspase3 antibody was purchased from Cell Signaling Technology, Inc. (Beverly, MA, USA). Primary polyclonal anti-P53, anti-Cyclin-D1 and secondary goat anti-rabbit, and goat anti-mouse antibodies were obtained from Santa Cruz Biotechnology, Inc. (Delaware Ave. Santa Cruz, USA).

*Plant Extract*

The gum of Baneh was collected from Jebalbarez Mountain (Kerman province, Southern part of Iran) in October. The voucher specimens (No. 2341) were deposited at the Herbarium of the Shahid Bahonar University of Kerman (Kerman, Iran). The collected gum was dried and powdered then dissolved in DMSO and used for treatment. The final percent of DMSO was less than 1%.

*Cell Culture*

MCF-7 (human breast adenocarcinoma cell line) cells were obtained from National Cell Bank of Iran (NCBI). The cells were grown with Dulbecco’s modified Eagle’s medium supplemented with 10% fetal bovine serum, penicillin (100 U/mL), and streptomycin (100 mg/mL). They were maintained at 37 °C in a 5% CO2 atmosphere. Growth medium was changed three times a week. The cells were plated at the density of 5000 per well in a 96-microplate well for the MTT assays. For protein extraction, the cells were grown in a 6-plate well and permitted to attach and grow for 24 h. Then the cells were incubated with different concentration of the gum alone or in combination with anticancer drugs doxorubicin.

*Cell Viability Analysis*

Cellular viability was assessed by the reduction of 2-(4, 5-dimethylthiazol-2-yl)-2, 5-diphenyltetrazolium bromide (MTT) to formazan. MTT was dissolved in PBS and added to the culture at final concentration of 0.5mg/mL. After additional 2 h incubation at 37 °C, the media were carefully removed and 100 µL DMSO was added to each well, and the absorbance (OD) values were determined by spectrophotometry at 490nm with microplate reader (Eliza MAT 2000, DRG Instruments, GmbH). Each experiment was performed 5-6 independent times. The results were expressed as percentages of control.

*Immunoblotting Analysis*

MCF-7 cells were homogenized in ice-cold buffer containing 10mM Tris-HCl (pH 7.4), 1mM EDTA, 0.1% SDS, 0.1% Na-deoxycholate, 1% NP-40 with protease inhibitors (1mM phenylmethylsulfonyl fluoride, 2.5 µg/mL of leupeptin, 10 µg/mL of aprotinin), and 1mM sodium orthovanadate. The homogenate was centrifuged at 14000 g for 15min at 4 °C. The resulting supernatant was retained as the whole cell fraction. 

Protein concentrations were measured using the Bradford method and equal amounts of protein (40 µg) were resolved electrophoretically on a 12% SDS-PAGE gel and then transferred to PVDF membranes (Hybond ECL, GE Healthcare Bio-Sciences Corp. NJ, USA). After overnight blocking at 4 °C with 5% nonfat dried milk in Tris-buffered saline with Tween 20 (blocking buffer, TBS-T, 150mM NaCl, 20mM Tris-HCl, pH 7.5, 0.1% Tween 20), the membranes were probed with antibody of caspase3 (1:1000 overnight at 4 °C), P53 (sc-126), Cyclin-D1 (sc-753) (1:1000, for three hours at room temperature). After washing in TBS-T (three times, each time 10 min), the blots were incubated for 60min at room temperature with a horseradish peroxidase-conjugated secondary antibody (1: 15000). All antibodies were diluted in blocking buffer. The antibody antigen complexes were detected using the ECL system and exposed to Lumi-Film chemiluminescent detection film (Roche, Germany). Lab Work analyzing software (UVP, UK) was used to analyze the intensity of the expression. β-actin immunoblotting (1: 1000) was used as loading control. The immunoblotting experiments for each protein were performed 3-4 independent times.

*Statistical Analysis*

The results are expressed as mean ± SEM. The differences in mean cell viability and blotting data between experimental groups were determined by one-way ANOVA, followed by Tukey test. 𝑃 < 0.05 was considered significant.

## Results

*The effect of pistacia atlantica gum on cell viability*

**Figure 1:**
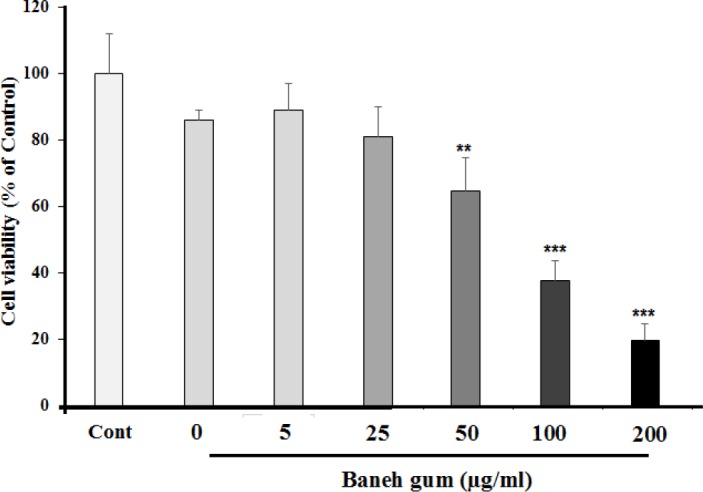
Effects of Baneh gum on MCF-7 cells viability. Cells were treated with gum for 24 h and then the viability was measured by MTT assay. Baneh gum reduced the cell viability, in dose dependent manner. Data are expressed as mean ±SEM; n = 6 wells for each group; **P < 0.01, and ***P < 0.001 significantly different versus control and vehicle treated cells

At first, MCF-7 cell viability was analyzed by MTT assay. After 24 h attachment/grow period, the cells were exposed to different concentration of *pistacia atlantica* gum (5, 25, 50, 100, and 200 µg/mL) for a 24 h period. Figure 1 shows that the gum could decrease MCF-7 cell viability in a dose dependent manner. The gum at doses of 100 and 200 µg/mL potently elicited cell death after 24 h and had a moderate effect in 50 µg/mL, while it could not prevent cell damage in dose of 5 and 25 µg/mL.

*The effect of combinational treatment of gum and doxorubicin on cell viability*

**Figure 2:**
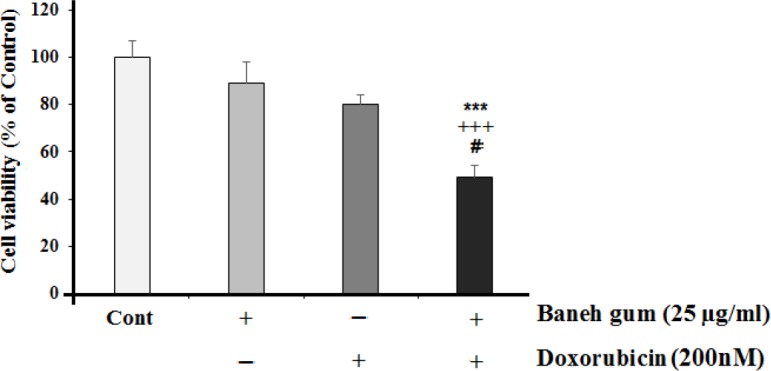
Effects of 25 µg/mL of Baneh gum alone or in combination with doxorubicin (200 nM) on MCF-7 cells. Combination treatment in comparison with each drug alone can induce significant cell toxicity. Each value in graph represents the mean ±SEM; n = 6 wells for each group; ***P < 0.001 significantly different versus control treated cells, +++*P *< 0.001 significantly different versus gum alone treated cells, #*P *< 0.05 significantly different versus doxorubicin alone incubated cells

For this reason, the non-effective dose of Baneh gum (25 µg/mL) was combined with doxorubicin. The data showed that doxorubicin at doses of 10 nM did not show a significant toxic effect on MCF-7 cells. However, combinational therapy of gum and doxorubicin (25 µg/mL and 200 nM respectively) significantly showed cytotoxic effect (Figure 2).

*The effect of gum alone or in combination with doxorubicin on caspase3, P53 and Cyclin-D1 protein level*

**Figure 3:**
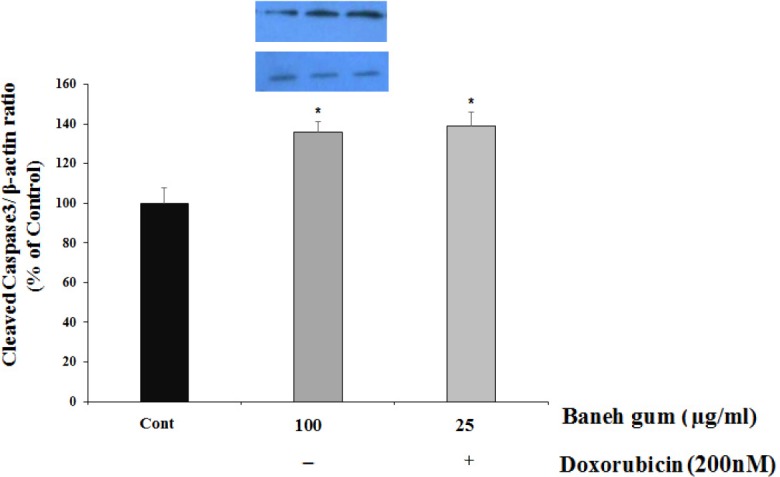
Effect of Baneh gum treatment alone and in combination with 200 nM doxorubicin on caspase 3 protein activation. Cells were incubated with gum alone (25 µg/mL) or in combination with doxorubicin (200 nM) for 24 h and then proteins were extracted and protein expression was measured by western blot. Each value in graph represents the mean ± SEM band density ratio for each group; ***P < 0.001 significantly different versus control treated cells. β-actin was used as an internal control

To examine the potential cellular mediators of Baneh gum induced cell damage, some protein markers of cell apoptosis (caspase3 and P53) and cell proliferation (Cyclin-D1) were evaluated in treated cells. In cells groups that had 100 µg/mL of gum alone and combination of gum (25 µg/mL) and doxorubicin (200 nM) for 24 h, the amount of cleaved caspase3 significantly increased as compared to control group (Figure 3).

**Figure 4:**
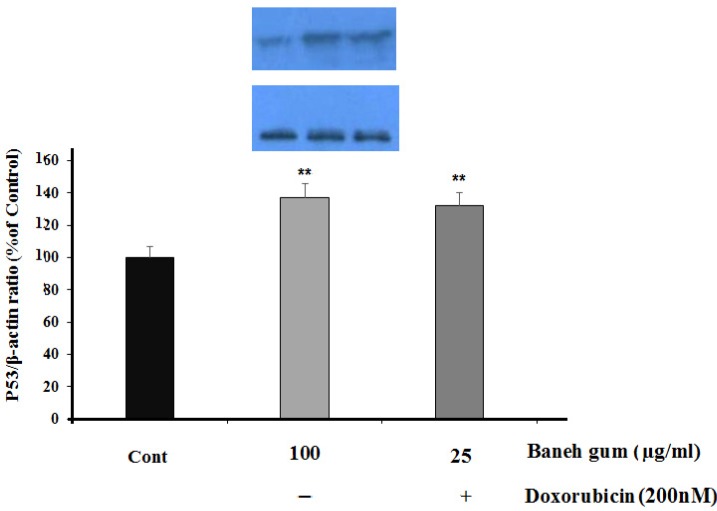
Effect of Baneh gum treatment alone and in combination with 1µg/mLl doxorubicin on P53 protein level. Cells were incubated with gum alone (25 µg/mL) or in combination with doxorubicin (200 nM) for 24 h and then proteins were extracted and protein expression was measured by western blot. Each value in graph represents the mean ±SEM band density ratio for each group; **P < 0.01 significantly different versus control treated cells. β-actin was used as an internal control

Furthermore, P53 protein was significantly increased in gum-treated cells (*P *< 0.01) and combination treatment (*P *< 0.01) as compared to control (Figure. 4).

**Figure 5:**
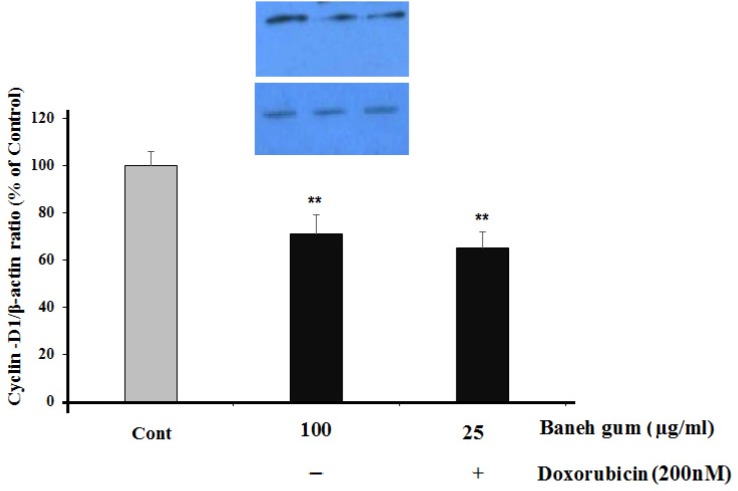
Effect of Baneh gum treatment alone and in combination with 1µg/mL doxorubicin on Cyclin-D1 down-regulation. Cells were incubated with gum alone (25 µg/mL) or in combination with doxorubicin (200 nM) for 24 h and then proteins were extracted and protein expression was assayed by western blot. Each value in graph represents the mean ± SEM band density ratio for each group; **P< 0.01 significantly different versus control treated cells. β-actin was used as an internal control

As shown in Figure 5, there was a significant decrease in Cyclin-D1 protein level in gum-treated (*P *< 0.01) and combination treatment (*P *< 0.01) cells (Figure. 5).

## Discussion

Cancer is a very complex disease which abnormal intracellular signal transduction is related to its occurrence and development (19). To date, chemotherapy is one of the main methods for cancer treatment and most agents used for this reason that have different unwanted side effects. Hence, recently, the research has been focused on effective chemo preventive agents with natural base that have high specificity and low toxicity on other normal cells. In this study, the gum of Baneh could decrease cell viability of MCF-7 cells. This effect is performed through the induction of apoptosis and inhibition of cell proliferation. 

Treatment strategies that can make the apoptotic signaling pathways reappear towards normality have the potential to decrease cancer cells. ROS are thought to play multiple roles in tumor initiation, progression, and maintenance. In addition to promoting role of ROS on cell growth under non-stress condition, ROS appears to activate and modulate apoptosis when cells are under stress. ROS levels are increased in the cells exposed to various stress agents, including anticancer drugs and they promote apoptosis by stimulating pro-apoptotic signaling component (20, 21). 

Caspase-3 plays a central role in executing apoptosis which is altered in most cancer cells. Skin extract of baneh has strong anti-proliferative activity by the activation of caspase-3 in T47D cells and which is more efficiently than doxorubicin (5). It has been shown that human breast cancer cells are damaged by baneh through its anti-proliferative property (7). Our result showed that baneh gum alone or in combination with doxorubicin could induce Caspase-3 activation. Difference between our result and the mentioned studies is the usage of different parts of baneh for such treatments (we used baneh gum and the other used skin of fruit). In addition, in our study the effective doses are tenfold fewer than earlier reports.

It is supposed that the anticancer activity of Baneh extract is due to its considerable amounts of polyphenolic compounds, flavonoids, and anthocyanin (9). It has been shown that the main components of *pistacia atlantica* gum are alpha (46.58%) and beta (9.08%) pinene respectively (22). Alpha and beta pinene have strong cytotoxicity toward human ovarian, hepatic and liver carcinoma cells (23). Furthermore, it has been shown that alpha pinene is able to induce apoptosis which is evidenced by early disruption of the mitochondrial potential, production of reactive oxygen species, and increase in Caspase-3 activity in melanoma cells (24). In human leukemic cells pinene reduces the protein expression of AKT and inhibits cell proliferation (25). Different studies have shown that alpha and beta pinene have antioxidant property (26, 27). In addition, baneh extract has pro-apoptotic, growth inhibitory, and cell cycle arrest property in HT29 cells (9). Those studies were done on pericarp extract of baneh and an effective dose was around 1 mg/mL but in our study baneh gum effectively induces apoptosis and cell arrest in 100µg/mL.

Cell growth in cancer cells was uncontrolled, whereas cell growth is normally controlled by cell cycle through Cdk and Cyclins. Cyclin-D1 is one of the critical biochemical switches in cell cycle (28). Up-regulation of this protein is documented in many cancers (29-31). It has been shown that gum mastic of *p. lentiscus* inhibits cell growth and blocks cell cycle in G1 phase and decreased expression of Cyclin-D1 and P-AKT protein level. Furthermore, these component increase the levels of tumor suppressor proteins (9). Surprisingly, the data showed that baneh gum alone or in combination of doxorubicin significantly reduces the expression of Cyclin-D1 in breast cancer cell line. Surprising, effective dose in our study was 10 fold fewer than that in mentioned studies.

P53 as a tumor suppressor regulates Maspin expression. Maspin is a serine protease inhibitor with tumor suppressive activity which is down-regulated in cancerous condition. (32-35). Maspin expression is directly regulated by the P53 gene. P53 induces maspin expression in prostate cancer cells and suppresses tumor growth and metastasis (36). It has been reported that gum mastic obtained from the stem and leaves of *Pistacia lentiscus* trees enhances maspin expression (37). Surprisingly, the data showed that P53 expression levels were significantly increased following baneh gum treatment alone or in combinational therapy group. 

Previous studies demonstrated that p53 protein has a key role in the regulation of Cyclin-D1 expression in different cancer lines. It has been shown that aberrant p53 expression occurs prior to the over-expression of Cyclin-D1, and this protein may elevate the over-expression of Cyclin-D1 to gain a growth advantage (38). It has been shown that one of the downstream effector of NF-κB is Cyclin-D1 and antitumor components such as curcumin could down-regulate cyclin-D1 due to NF-κB inhibition in tumor cells (39). Alpha pinene inhibits the nuclear translocation of NF-κB due to up regulation of NF-κB (40). G2\M shift is controlled by Cyclin-B1\CDC2 complex in cells and different factor such as P53 regulate the activity of this complex (41, 42). 

Although the anticancer effect of baneh and baneh gum have been previously reported but in this study the causality of lower doses of baneh gum may be due to difference of weather condition in jebalbarez than west area of Iran commonly used for baneh collection. 

In this study baneh gum potentiated the effect of doxorubicin in MCF-7 cells. So, synchronized treatment of doxorubicin with baneh gum may be considered as a new strategy for decreasing doxorubicin doses and its side effect which routinely occurs with high doses of doxorubicin. Overall, this study demonstrated that baneh gum extract has a potential anti-proliferative and anti-apoptotic property in MCF-7 cells and can be used as pharmaceutic case study for breast cancer treatments but further studies are necessary investigation of its mechanism in detail.
